# Health-related integration interventions for migrants by civil society organizations: an integrative review

**DOI:** 10.1080/17482631.2021.1927488

**Published:** 2021-05-30

**Authors:** Pelle Pelters, Eva-Carin Lindgren, Catrine Kostenius, Marie Lydell, Krister Hertting

**Affiliations:** a School of Health and Welfare, Halmstad University, Halmstad, Sweden; b Department of Education, University of Stockholm, Stockholm, Sweden; c Department of Food and Nutrition, and Sport Science, University of Gothenburg, Gothenburg, Sweden; d Division: Health and Rehabilitation, Department of Health Sciences, Luleå University of Technology, Luleå, Sweden; e Norrbotten Association of Local Municipalities, Luleå, Sweden

**Keywords:** Acculturation, civil society, health, integration, integrative review, intervention

## Abstract

**Purpose**: Migrants are a vulnerable group concerning health and integration. Civil society organizations are deemed important for the integration and health of migrants and have been encouraged to help. This study explored health and acculturation, as expressed in research into health-related integration interventions for migrant groups provided by civil society organizations.

**Methods**: Databases, journal websites and reference lists were searched in an integrative review. Thirteen articles were found and analysed using concepts of health strategies/perspectives and of acculturation with regard to general and health culture.

**Results**: Studies were divided between two primary spectrums: 1) assimilation-integration and 2) integration-separation spectrum. Spectrum 1 interventions tend to promote assimilation into the present host culture and into a Western view of health. They are mostly driven by representatives of the host culture. Spectrum 2 interventions are characterized by a greater openness concerning the home-culture understandings of health, alongside an appreciation of the home culture in general. They are mostly migrant-driven.

**Conclusions**: The different acculturating approaches in migrant and native-driven civil society organizations call for an awareness of their guiding health and acculturation assumptions and their applied perspectives and strategies. This awareness is considered crucial in order to achieve desired health and acculturation outcomes.

## Introduction

Even though migration for economic, political and social reasons has always been a part of global society, conflicts in the world have put migration policies in focus in many countries in recent years. After major migration and displacement events, a total of 3.5% of the world’s population are international migrants, which already exceeds projections made for the year 2050 (International Organization for Migration [IOM], 2019). According to UNHCR (2018), 68.5 million people have been forcibly displaced from their homes. Although those countries neighbouring ‘trouble spots’ are often the first to receive large numbers of refugees, non-neighbouring Western countries are affected as well.

In the West (as the focus of this study), welfare systems are put under pressure (Esses et al., 2017). Among those who fall under the spotlight and receive encouragement from the government to become involved in the integration of migrant groups into society (cf. e.g., Agergaard, 2011; Ambrosini & Van der Leun, 2015; Fredriksson et al., 2018; Swedish Government, 2010) are organizations belonging to what is known as “civil society”. Civil society can be understood as a “collective term for the sphere in democratic societies that consists of voluntary associations between individuals (popular movements, different types of associations, religious denominations, etc.) that are independent of the state. Civil society is a link between the private and the public and is an arena for interaction outside the state and the market.” (Nationalencyklopedin, 2021, our translation). International research confirms that civil society organizations can be essential for accessing social capital and therefore play a compensatory role for economically and socially disadvantaged groups (Field, 2005; Portes & Rumbaut, 2001; Zhou & Kim, 2006). In Canada, a policy focusing on multiculturalism and the enablement of civil society has emphasized the agency of ethno-cultural communities (Schmidtke, 2018). This has contributed to making diversity and cultural pluralism principal issues of public debate and civil society organizations have assumed an important role as political advocates for migrants.

When the state increases restrictions on immigration, civil society has come to play an increasingly important role in handling migrant groups under challenging situations (Ambrosini & Van der Leun, 2015). On the one hand, civil society can function as a form of advocacy for migrants. On the other hand, few civil society organizations challenge the basic assumptions of government policy. Although these organizations usually claim to promote an exchange-oriented integration of migrants, they may indeed facilitate an incorporating assimilation (Ambrosini & Van der Leun, 2015). Furthermore, referring to the case of sports organizations as one branch of the civil society organizations aiming (among other things) at assisting migrant populations to integrate into society, Osterlund and Seippel (2013) argue that social integration is unevenly distributed among different groups and civil society also has varying capabilities of promoting social inclusion. The conclusion is that, even though researchers have some critical remarks on the role of civil society in the process of migrant integration, civil society could make a positive contribution to the migration process.

“Migrant groups” as a term has been used to represent a large variety of people concerning different time frames of residence, reasons for migrating and legal status reflected in designations such as “immigrants”, “newcomers” and/or “refugees” (Hannigan et al., 2016). All of them face specific challenges and different experiences. Still, they are united by the all-encompassing inevitability of developing a standpoint towards new cultural conditions and expectations and the demand to deal with these. These specific life circumstances pose an interactionist identity challenge (Burr, 2003).

Moreover, migrants belong to one of society’s most vulnerable groups, in which issues of health inequalities are at stake (Esses et al., 2017). Research shows that refugees can suffer from physical and mental health problems (Lecerof et al., 2016; Manesis, 2014) and that new arrivals may end up being excluded when they enter a new society (Viruell-Fuentes et al., 2012). In a study on discrimination and migrant health, Nakhaie and Wijesingha (2014) discovered that feelings of discrimination led to poorer perceived health, especially among migrant women. According to Lecerof et al. (2016), mental illness is more common among migrants than among other groups of people in Europe. Shawel Abebe et al. (2014) argue that mental illness in refugees can be exacerbated by a series of adverse events, such as trauma and acculturative stress. They claim that health varies greatly depending on socio-cultural and economic contexts, gender, generation and social integration. Mental illness has been shown to reduce quality of life (Baños et al., 2017) and the health of new arrivals has been a major challenge in many countries (Shawel Abebe et al., 2014). This is not surprising as transferring from one country to another is a complex process.

However, questions about the health of new arrivals are intricate. Kwak (2018) and Kennedy et al. (2014) discovered the existence of the healthy migrant effect (HIE), which states that health, in general, can be better among new arrivals than the health of host society citizens and long-term migrants. According to Kwak (2018), new arrivals were in better health but suffered from more stress regarding adaptation. This changed over time, and for long-term migrants in general the situation was the opposite (and similar to the situation of host society citizens). Thus, health and well-being among migrants involve a dynamic and multi-layered process, which may be questioned or even compromised by the multidimensional effort in balancing identity when encountering a new society (Strandbu, 2005; Walseth, 2006; Zacheus, 2010). Developing a new migrant-identity that is based on contribution and self-esteem may, however, act as a counterbalance to less favourable tendencies that might add up to HIE. Hence, a successful integration that promotes a process towards “being an active, integrated part of society” instead of just “being in society” may promote health and the continual negotiation of a new identity in the host society.

There have been a limited number of review studies on health-related interventions with migrants, but none of these review studies were focused on interventions by civil society. Three studies focused on health-related interventions with specific ethnic groups (Organista, 2009; Stein & Guzman, 2015; Yong et al., 2016) and two studies focused on community-based health interventions (Gibbs et al., 2014; Williams & Thompson, 2011). Community-based interventions could include civil society. However, the concept was used in a broader sense in these two studies. Therefore, we regard the lack of review studies on health-related integration interventions in civil society to be a knowledge gap and find it relevant to conduct a review of those interventions for migrant groups provided by civil society organizations. Health-related integration interventions are here understood as interventions that address issues of both health and integration.

## Aim and research questions

The aim of the study was to explore the aspects of acculturation which are expressed in research into health-related integration interventions for migrant groups provided by civil society organizations. To achieve this aim, we studied on the one hand how civil society’s health-related integration interventions are oriented towards acculturation strategies in migrant groups and society at large. On the other hand, we investigated which health perspectives and health strategies are constructed in civil society’s health-related integration interventions.

## Theory

We used a dual theoretical approach in order to answer the research questions posed and cover the aim of the study through its twin focus on integration and health.

To understand the specific integrative approach used in different interventions, we turned to a model developed by Berry (1997, 2008) to expose processes associated with migration and encounters between a dominant, i.e., home, and non-dominant or host culture in a society. Although we are well aware of the situatedness, ambiguity and ever-changing diversity of cultures locally and in different group-settings, following Berry (2008) we use the term “culture” in the singular. Using “culture” in that way emphasizes the contact between people and concepts rooted in different cultural contexts, the “dominant group” and several “non-dominant groups” with their respective “heritage cultures” that Berry (2008, p. 330) mentions. By doing so, the terms “home” respectively “host” culture refer to the locally and personally valid cultural contexts and interpretations that are present in the specific encounters which individuals experience.

This being said, Berry describes intercultural strategies used in acculturation, which itself is understood to be a two-way process associated with complex processes of continuity and change in people’s lives in a new society. The processes are affected by, for instance, migration motives (e.g., immigration, refugee, voluntariness, mobility and permanence), the political context of the society of origin and the society of settlement, as well as individual factors and social support (Berry, 1997).

Berry (1997) describes four types of acculturation strategy when entering new societies: integration, assimilation, separation/segregation and marginalization. These strategies deal with issues of cultural maintenance and participation in the society of settlement and are not merely the result of individual choices but are influenced by the above-mentioned processes. Integration occurs when a degree of cultural integrity is maintained while simultaneously seeking to be an integral part of larger social networks. Assimilation refers to adjustment to the dominant culture and repression of one’s own culture of origin. Separation refers to maintenance of the culture of origin and not connecting to the society of settlement, while marginalization refers to a lack of contact with both the society of origin and society of settlement. According to Berry (2008), the corresponding strategies of larger society at large are multiculturalism, melting pot, segregation and exclusion. For integration to occur, the societies of settlement must be characterized by the acceptance of values of cultural diversity and multiculturalism (Berry, 1997, 2008; Esses et al., 2017).

Berry’s model of acculturation has been widely utilized, but also criticized. One criticism is that acculturation is more complex than fixed categories and individuals’ “free” choices of acculturation strategies (Ward & Geeraert, 2016; Weinreich, 2009). Furthermore, the concepts of acculturation have been criticized for supporting an underlying and uncritical process of assimilation to the host culture (Weinreich, 2009). However, Berry (2009) illuminates how integration has a specific meaning in the model, which distinctively differs from assimilation and argues that integration is supported in societies characterized by multiculturalism and intercultural relations. The critical remarks have been considered in this study and Berry’s acculturation strategies are used as lenses from a host culture perspective serving as descriptive spectrums rather than fixed functional outcomes of immigration.

Berry’s terminology is used in two ways for the description of acculturation in the data material about integration interventions, one of which concerns culture in general and hence the way Berry (1997, 2008) himself applies his acculturation strategies, while the other concerns a Western health culture. To specify what is meant by Western health culture, we refer to Lupton (2012) as a starting point. What the author described concerning medicine, we deem valid for health in general: “‘culture’, more broadly, should be understood as the conglomeration of meanings, discourses, technologies and practices that accumulate around medicine *within* Western societies as well as outside them” (Lupton, 2012, p. viii). This Western health culture implies a hegemonic view, which is characterized by biomedical, technoscientific interpretations of health statuses grounded in a logic of evaluation defined by the natural sciences (i.e., based on quantitative data, rationality, objectivity, etc.). This approach towards evaluation is combined with an individual and responsibilising perspective regarding the production and producibility of health, which is, moreover, generally goal-oriented, according to scholars in the field of sociology of health and illness (Clarke et al., 2003; Nettleton, 2013).

This hegemonic point of view of health in Western societies may therefore be regarded as both a cultural means and a marker of integration (Ager & Strang, 2004), which will be used to describe the interventions’ adherence to this culturalised approach. Please note that, as a consequence, we apply a discursive and not a dimensional view of health. Moreover, we act on the assumption that Berry’s (2008) acculturation strategies may be used to describe culture in general and health culture in particular. We primarily apply the spectrum of integration, assimilation, separation/segregation and marginalization and to some degree that of multiculturalism, melting pot, segregation and exclusion, if possible. Our intentions were to add an understanding of the options regarding the adherence to health as an expression of a culture which may be of host-like or home-like character and, as such, incorporated in the different interventions which could be identified in our study.

To ascertain the interventions’ support of the health(y) development of migrant groups, the interventions’ way of dealing with the perspectives and strategies used in health interventions was determined. We distinguished between prevention and a promotion strategy as well as a pathogenic and a salutogenic perspective in health interventions by examining the ways of dealing with health issues in the intervention.

According to Antonovsky (1996, 1993, 1979), the dominant logic of the pathogenic perspective is characterized by a focus on problems, diseases, obstacles and deficits. The perspective is driven by a motivation to identify embodied, behavioural and ecological risks as well as maleficent developments in and towards disease and to delete or at least alleviate these by applying a problem-solving mode. The prevention strategy, which is often linked to this pathogenic logic, is therefore based on the notions of risk and protection and tends to adopt an avoiding course of action to keep a specific, negatively-labelled outcome at bay (Antonovsky, 1996, 1993, 1979).

The logic of the salutogenic perspective, on the other hand, focuses on the origin and determinants of health instead of disease. Resources considered beneficial for promoting health at the individual, family, and community level (both generalized and specific resistance resources) are at the centre of attention. Their identification makes it possible to capitalize on aspects that function well and improve them even further (Antonovsky, 1996, 1993, 1979). The World Health Organization introduced health promotion in the Ottawa Charter as the process of enabling people to increase control over, and to improve, their health (World Health Organization [WHO], 1986). The promotion strategy, often following in the footsteps of salutogenesis, is therefore based on the notion of potential as well as development and is devoted to the enhancement of resources that are labelled positive or desirable as the preferred course of action. The strategies of prevention and promotion, as well as the logics of pathogenesis and salutogenesis are today fundamental in health work, with their main features (as presented and applied in this paper) being well established and largely uncontested (Mittelmark et al., 2017). Prevention, promotion, pathogenesis and salutogenesis have been used to determine the strategic approach of the interventions in the chosen articles.

## Materials and method

### Integrative review

An integrative review is used in this paper because it is the broadest of the review methods. An integrative review permits both quantitative and qualitative studies and reference to both theoretical and empirical literature with a wide range of aims and concept definitions (Torraco, 2005; Whittemore & Knafl, 2005). Its purpose is to review methods, theories, and/or empirical studies concerning a particular topic and it differs from other forms of literature review in that the former enables a broader inclusion of data and the latter largely examines studies that apply similar methodologies (Whittemore, 2005). As health-related civil society integration interventions have been scarcely researched—see selection process below—the method was chosen because of its broad focus in order to include the widest possible variety of studies.

### Data collection and selection

As this paper describes a review study, which used published research articles as data, there was no need to apply for ethical approval. A sample of scientific articles has been collected by applying a set of inclusion criteria to three different search domains: databases, selected scientific journals and reference lists. The inclusion criteria comprised peer-reviewed full-length articles that were written in English and published between 2007 and 2017. Regarding content, these articles describe the work of civil society organizations directed towards migrant groups, aiming to promote the inclusion of these groups into the host society by impacting on their health. All other hits/articles were excluded as they did not match the study aim.

The first search domain covers a relevant selection of scientific databases: PubMed, Academic Search Elite, Eric, SportDISCUS and PsycInfo, which were systematically searched in November and December 2017. These databases were searched using the following keywords and strategies, summarized here as a Boolean search mode: i) Acculturation OR Integration AND ii) Health OR Health promotion OR Prevention AND iii) Refugees OR Immigrants OR Newcomers AND iv) Programme(me) OR Intervention OR Support Group.

Different migrant groups were equally included in the search (refugees, immigrants, newcomers). Although we know that different categories of migrants need to deal with different migration-related challenges and experiences, we made the decision to include refugees, immigrants and also newcomers, due to their similar challenge of facing (a) new culture(s). We used a broad definition of migrants as “any person who is moving or has moved across an international border or within a State away from his/her habitual place of residence, regardless of (1) the person’s legal status; (2) whether the movement is voluntary or involuntary; (3) what the causes for the movement are; or (4) what the length of the stay is” (IOM, 2018). Moreover, the search term “civil society” was tested and then discarded at this point of the search process as it did not result in any hits. Instead, we decided to apply this criterion to the screening stages.

At this point, a second search domain (specified scientific journals) was added to enhance the data collection. The journals were selected because of their relevance to the research field and focus and comprised the International Journal of Migration, Health and Social Care, the Journal of Immigrant and Minority Health, Social Inclusion, as well as the Journal of Immigrant Health. Here, the search focused on the keywords “intervention”, “support group” or “programme” and “civil society”, as the journals already covered aspects of health and migration. The resulting 30 records identified were added to the title sorting process.

**Figure 1.**
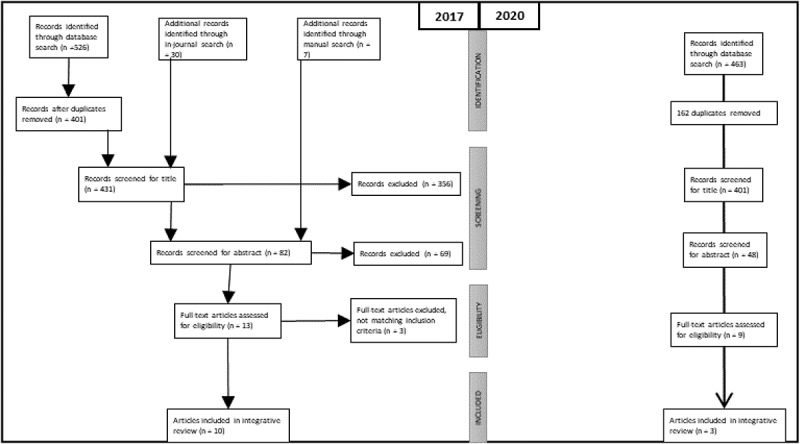
Flow of information through the different phases of the integrative review 2017 and 2020 (Moher et al., 2009)

The subsequent screening process focused on the inclusion criteria of finding interventions, support groups or programmes that were conducted by civil society organizations. The screening included both a title and abstract sorting, leading to a total of 10 articles that met the inclusion criteria. The reference lists of these 10 articles were manually searched to add further relevant articles to the data material. After their abstracts were screened, three of the seven articles that could be identified manually proved to fit the inclusion criteria. Finally, a total of 13 articles were read at full length in order to determine their eligibility. A final sample of 10 articles (three case presentations, one conceptual paper and six empirical articles on nine interventions or programmes) was included in the integrative review. The search process—including identification, screening, eligibility and inclusion processes—has been illustrated in a flowchart (Figure 1), which has been arranged according to the PRISMA flow diagram (Moher et al., 2009).

In June 2020, the database search was repeated to update search results. Due to accessibility issues, Scopus, PsychInfo and Pubmed were chosen to be screened using the same keywords, search strategy and criteria for inclusion and exclusion as in 2017, but focussing on the publication period 2017–2020. A total of 463 hits were found. After removing 162 duplicates, hits were screened by title (48 remaining hits), by an abstract (nine remaining hits) and by full-text. A final sample of three articles was in the search in June 2020 added to the existing body of data, making the total number of articles in this review 13.

### Analytical procedure

The selected articles were initially read to gain an overview of their content. Each article was then inserted into a table that covered author, year of publication and title, objectives of the article and design of the study, civil society organization, intervention location and participants, as well as a brief description of the intervention (see Table I for an overview of the research articles included, listed according to authors’ names in alphabetical order). A detailed summary of the intervention was also written.

**Table I.** Overview of included articles

Author (year) title	Type and objectives of the article	Civil society (CS) agent and place	Participants	Intervention
Akinsulure-Smith, AM & Jones, WL. (2011) Nah We Yone—a grassroots community-based organization in New York city: successes, challenges and lessons learned. International Journal of Migration, Health and Social Care, 7(1): 44–57.	Presentation of intervention Objectives: -discuss the development of a grassroots, community-based support organization, “Nah We Yone” (NWY) -review the organization’s background, the challenges faced and the services available	CS agent: Grassroots community-based organization Place: New York City area	Forced migrants of African origin with a history of refugee trauma, war, and human rights abuses	Community-based empowerment model with culturally informed social and psychological programs: -detainee-support program; therapeutic programs; internship program at NWY for community members; educational services and training (internally and externally); advocacy Drawing on relationality and social networks; Aware of African people’s reluctance to seek formal mental health services and rely on traditional healers, religious leaders, elders, and family members
Birman, D.; Beehler, S.; Harris, E.M.; Everson, M.L.; Batia, K.; Liautaud, J.; Frazier, S.; Atkins, M.; Blanton, S.; Buwalda, J. (2008) International Family, Adult, and Child Enhancement Services (FACES): A Community-Based Comprehensive Services Model for Refugee Children in Resettlement. American Journal of Orthopsychiatry, 78(1): 121–132.	Case study Objectives: to provide the first detailed description of the clientele, program and impact of a mental health program (FACES) serving a diverse group of refugee children and adolescents	CS agent: Heartland Alliance for Human Needs and Human Rights (Charity) with extensive history of providing services for refugees and immigrants Place: USA	Multicultural and multilingual group of refugee children, adolescents and their families	International Family, Adult, and Child Enhancement Services (FACES): culturally-sensitive, community-based mental health program with comprehensive care perspective and service spectrum: counselling, different forms of therapy for individuals, groups, families; psychiatric services; consultations; support - customized case management: basic survival and adjustment prior to traditional mental health service -multidisciplinary and multi-ethnic team
Block, K., Gibbs, L. (2017) Promoting Social Inclusion through Sport for Refugee-Background Youth in Australia: Analysing Different Participation Models. Social Inclusion, 5(2): Pages 91–100.	Exploratory qualitative study Objectives: -report 3 available models of sports participation in different interventions -clarify implicit assumptions and conceptual frameworks -examine disadvantages, benefits, challenges from the point of view of the provider -discuss the models’ impacts and capacity to promote sustainable engagement & social inclusion	CS-agent: different agent/providers of sport (NGOs, local governments, schools, sports clubs [elite, grassroots]) Place: Melbourne, Australia	Young people from refugee backgrounds	3 models in 10 different programs A: short term, B: ongoing programs directed at youth with a refugee background, C: integration into mainstream clubs -perceived benefits: teamwork, being focused, working hard/ commitment, respect, fun, health, learning leadership, goal setting, positive self-image, positive male role model offered (coach/mentor) enhanced social capital participants perceived as learning to apply values and skills learned from sport to other aspects of life
Dhillon, K. K., Centeio, E. E., & Dillon, S. (2020). Drumming and dancing: creative movement for Convention refugee youth in a physical activity space. Sport, Education and Society, 25(3), 318–331.	Ethnographic study Objective: to capture Convention refugee youths’ movement narratives within an organized, community education, physical activity space (focus on drumming and dancing) as a space through which acculturation can be fostered	“Irene’s project”: Free, recreational after-school club (community education), each school day for 24 weeks Southwestern Ontario, Canada	17 young Convention refugees (11–18 years of age) with different migration backgrounds (home cultures)	Provision of a variety of dance, music and other artistries, including a drumming circle as a self-created space (i.e., programming not initially intended to be drumming and creative movement) Facilitated by a physical activity leader who sought to unlock the youth’s creative power by encouraging self-expression and the mutual teaching of rhythms & moves
Gerber, M. M., Callahan, J. L., Moyer, D. N., Connally, M. L., Holtz, P. M., & Janis, B. M. (2017). Nepali Bhutanese refugees reap support through community gardening. International Perspectives in Psychology: Research, Practice, Consultation, 6(1), 17–31. http://dx.doi.org/10.1037/ipp0000061	Evaluative mixed-methods study Objective: to explore differences in experiences among Bhutanese refugees resettled in the USA who do versus those who do not participate in community gardening as a supposedly mental health promoting intervention	CS agent: refugee resettlement agencies as provider (9 US NGO partners, 6 denominational, 1 developed from community-base, 2 general humanitarian, cf. https://www.acf.hhs.gov/orr/resource/resettlement-agencies) USA	Nepali Bhutanese refugees	Community gardening plots that are provided, supported and distributed by refugee resettlement agencies multiple plots of earth cultivated by different families or individuals, plus communal plots for shared crops of their choice aim: promote psychological healing, self-sufficiency, community engagement, a return of human dignity result: strengthened relations, get social support, feel like being back in Bhutan in the garden, improve independence & self-efficacy
Hála, J. (2017). The contribution of the German organization “Evangelisches Jugend- und Fürsorgewerk” in addressing immigration issues. Kontakt, 19(4), e304-e310.	Presentation of intervention Objectives: -description of good practice examples -to motivate Czech university students to gain personal experience from abroad for future effective solutions of immigration problems at home	CS agent: EJF = Evangelisches Jugend- und Fürsorgewerk Christian charity based on saving needy sufferers Article focus: Berlin, Germany (EJF owns several facilities in Germany, Poland, Czech Republic)	Legal and legally-living immigrants in different phases of introduction to the German society	Various social, educational, therapeutic projects, matching the immigrant’s phase of introduction to the German society: -incoming: stabilization and accommodation (mental, medical, physical) -orientation: social, legal and crime-preventive counselling & education of & on the host country, language classes -integration: counselling and education (exemplified in a family Centre setting, focus on pregnancy, family life, healthy living)
Gonzalez, A.; Lord, G.; Rex-Kiss, B.; Francois, J.J. (2012) Parents Beyond Borders: A Social Group Work Curriculum for Supporting Immigrant Parents and Building Solidarity. Social Work with Groups, 35(1): 18–34.	Presentation of intervention Objectives: Present the “Parents beyond borders” curriculum and its results	Neighbourhood-based community organization “Center for Family life” Place: Sunset Park, Brooklyn, New York, USA	Immigrants in this specific low-income neighbourhood in Brooklyn	13-week support group for immigrant parents to explore their social identity, reduce social isolation, build solidarity, become empowered/creative/critical, develop activism and connect with social services Focus on sharing acculturation stress and immigration trauma, experiencing empathy, validation, solidarity Background in trauma and family systems theory; narrative, art & drama therapy, social group work
Hashimoto-Govindasamy LS, Rose V. (2011) An ethnographic process evaluation of a community support program with Sudanese refugee women in western Sydney. Health Promotion Journal of Australia, 22(2): 107–112.	Ethnographic evaluation Objectives: Evaluate community-support program with focus on women’s perceptions of the program and ongoing settlement needs	- Penrith Women’s Health Center (charity) & NSW Sport and Recreation in cooperation with Sisters of Mercy; - Sydney, Australia (Mamre Homestead);	Sudanese refugee women	Exercise program within broader resettlement support program -introduce participants to physical activity and encourage regular exercise through structured and spontaneous activities: e.g., walking, dancing, Sudanese children’s games, beach day -time for socializing
Mwanri, L. Hiruy, K. Masika, J. (2012) Empowerment as a tool for a healthy resettlement: a case of new African settlers in South Australia. International Journal of Migration, Health and Social Care, 8(2): 86–97.	Conceptual paper Objectives: -empowerment strategy description -presentation of African Organization in South Australia (ACCSA) as an example of an empowering organization	CS-agent: Grassroots community-based ACCSA Place: South Australia	ACCSA represents 42 organizations and African communities from 18 sub-Saharan countries	-Various interventions, programs and advocating for settlement; education; employment and training; family values; family, gender roles and women; housing; identity and integration; law, police and social order; leadership and governance; local government and participation, health/prevention Health intervention: e.g., “Physical activity and nutrition program”, “cervical awareness project”, project against binge drinking, health care access information Multiculturalism: festival display of culture, etc.
Rich, K.A., Misener, L. & Dubeau, D. (2015) “Community Cup, We Are a Big Family”: Examining Social Inclusion and Acculturation of Newcomers to Canada through a Participatory Sport Event. International Journal of Migration, Health and Social Care, 8(2): 86–97.	Case study Objectives: -examine potential of sport in playing a role in social inclusion for newcomers to Canada, -examine participants’ experiences in this sport event and the potential of sport events to play a role in transition/ acculturation of newcomers -suggestions for sport event managers to improve event design and implementation	CS-agent: Catholic Centre for Immigrants, a community-based charity Place: Ottawa, Ontario, Canada	Newcomers (recent immigrants and refugees) as players and organizers	Soccer tournament: participatory annual sport event with immigrant contributions to sports and event organization Organization: -20 mixed event planning teams comprising newcomers and locals working on common organizational tasks - organization culture: welcoming, including, friendly, non-judgemental Sports during the tournament: - rules adjusted in order to promote a welcoming and inclusive atmosphere instead of competition: “spirit points” for playing style and attitude just as crucial to win as it is to score goals Participants perceive Cup as lowering the threshold to access sports; common interest linking different people; gaining Canadian experience (learning norms); meeting people from different cultures
Spaaij, R. (2012) Beyond the playing field: Experiences of sport, social capital, and integration among Somalis in Australia. Ethnic and Racial Studies, 35(9): 1519–1538.	Ethnographic study Objectives: - explore the role of recreational sport as a means and a marker of integration by analysing the lived experiences of Somalis from refugee backgrounds through sport -examine the extent to which participation in sport contributes to Somali’s social capital	CS agent: grassroots initiative, community-based soccer club Place: north-western suburb of Melbourne	African Australians with refugee background (mostly Somali)	Melbourne Giants FC: -regular soccer club: 60 paying members -established in late 1990s to provide sporting opportunities to African, particularly Somali refugees -sport as a meaningful and enjoyable leisure activity that provides a pathway into the host society and is free from clan politics: internal cohesion of Somali community promoted
Whitley, M.A., Coble, C., Jewell, G.S. (2016) Evaluation of a sport-based youth development program for refugees. Leisure/Loisir, 40(2): 175–199.	Exploratory evaluation of a sport-based program for refugee youth Objectives: assess participants’ perceptions and experiences in this program with reference to the acculturation framework	“Refugee Sport Club” (RSC) held at a non-profit organization, presented as a private initiative of the first author (charity) Place: Lansing, Michigan, USA	Refugees coming from African countries 2 age groups: 8–12 & 13–18 years	Sport-based youth development program based on Western Teaching Personal and Social Responsibility (TPSR) Model: progressive development of respect, teamwork, leadership, transfer to everyday life, each club meets 1 hour each week with a program duration of 8–10 weeks Meetings include: relational time; awareness talk on respect/ teamwork/ leadership; physical sports activities (soccer, basketball, volleyball—to broaden knowledge of “American” sports); group meeting; reflection time; final relational time Mentors: promote good decision making and personal development by focusing on individuals and asking questions instead of telling directly Participants’ appreciation of Western-style apparent
Whitley, M.A. & Gould, D. (2011) Psychosocial Development in Refugee Children and Youth through the Personal–Social Responsibility Model. Journal of Sport Psychology in Action, 1(3): 118–138.	Presentation of intervention Objectives: -describe sports program for refugee children and youth based on the TPSR Model. Presentation of program goals as well as strategies and procedures utilized in the implementation of the program [description of (9)]			

Acculturation strategies, according to Berry (1997, 2008), were used to identify the strategy or range of strategies applied to support the integration goal of the reviewed interventions. To explore the aspect of integration, the articles were sorted using the following steps: a) each intervention and the roles and reactions of the civil society organization that conducted the intervention and of the participants were compared to the four different acculturation strategies insubordinate and dominant groups in society (to the extent permitted by the content of the articles); and b) the articles were sorted using the spectrum of different strategies outlined by Berry’s (2008) model.

To determine the interventions’ support of health(y) development, a two-fold composite approach describing health strategies and the Western hegemonic view of health as presented in the “theory” section was adopted in order to sort the research articles. The practical sorting procedure concerning this two-fold health evaluation approach was conducted in two steps: First, the interventions were compared to strategies of prevention and promotion, as well as pathogenic or salutogenic perspectives as the background to these strategies. Second, the interventions were evaluated in relation to the Western hegemonic view of health and the motivation for the categorization of particular interventions was written.

Table II illustrates an overview of the analytic findings, taking into consideration: a) the (implicitly or explicitly) suggested acculturation strategies according to Berry (2008); b) the chosen health strategy of the intervention (prevention or promotion); c) the background perspective on the health of migrant groups (salutogenic or pathogenic); and d) the origin of those organizing the interventions (either native-driven, i.e., grounded in the host culture, or migrant-driven, i.e., organized by [former] migrants or refugees). The process resulted in a description of different spectrums concerning acculturation and health discourses, as well as health work. The term “spectrum” has been used to illustrate that there are tendencies but no clear-cut classifications.

**Table II.** Overview of findings

Author(s)	Acculturation	Health perspective	Health strategy	Organization management
Hála, 2017	assimilation	pathogenic	prevention	native-driven
Birman et al., 2008	assimilation—integration	pathogenic	prevention	native-driven
Block & Gibbs, 2017	assimilation—integration	salutogenic	promotion	native-driven
Hashimoto-Govindasamy & Rose, 2011	assimilation—integration	pathogenic	promotion	native-driven
Mwanri et al., 2012	assimilation—integration	pathogenic	prevention	migrant-driven
Whitley & Gould, 2011 + Whitley et al., 2016	assimilation—integration	pathogenic	promotion	native-driven
Gonzalez et al., 2012	integration	pathogenic	promotion	migrant-driven
Dhillon et al., 2020	integration	salutogenic	promotion	migrant-driven
Akinsulure-Smith & Jones, 2011	integration—separation	salutogenic	prevention	migrant-driven
Spaaij, 2012	integration—segregation	salutogenic	promotion	migrant-driven
Gerber et al., 2017	Integration—separation	pathogenic	promotion	native-driven
Rich et al., 2015	assimilation—integration—separation	salutogenic	promotion	native-driven

## Findings

13 articles have been identified that describe 12 different interventions or initiatives. The articles included four qualitative case studies (Birman et al., 2008; Dhillon et al., 2020; Rich et al., 2015; Spaaij, 2012), four interventions presented as practice examples (Akinsulure-Smith & Jones, 2011; Gonzalez et al., 2012; Hála, 2017; Whitley & Gould, 2011), three programme evaluations (Gerber et al., 2017; Hashimoto-Govindasamy & Rose, 2011; Whitley et al., 2016), one conceptual paper that used an intervention programme as a clarifying example (Mwanri et al., 2012) and one exploratory study describing different programmes (Block & Gibbs, 2017).

The interventions comprised four sports-based programmes (Block & Gibbs, 2017; Rich et al., 2015; Spaaij, 2012; Whitley et al., 2016/Whitley & Gould, 2011), two support services (Birman et al., 2008; Gonzalez et al., 2012), one exercise programme that added to a broader resettlement support programme (Hashimoto-Govindasamy & Rose, 2011), two programmes aiming for support through physical activity (Dhillon et al., 2020; Gerber et al., 2017) and three comprehensive programmes covering different areas of intervention. The first comprehensive programmes included a detainee-support programme, therapeutic programmes, an internship programme, educational services and training as well as advocacy (Akinsulure-Smith & Jones, 2011). The second comprehensive programme included activities and programmes concerning health, education, employment and training, family values, family, gender roles and women, housing, identity and integration, law, policy and social order, leadership and governance, as well as participation in local government (Mwanri et al., 2012). The third comprehensive programme provided informational, counselling and therapeutic services as well as addressing basic needs of refugees through a range of different activities and facilities provided by one charity organization (Hála, 2017).

The interventions are grouped below in accordance with Berry’s acculturation model (1997, 2008). In addition, summaries of the interventions’ health affiliations are provided at the end of each strategy spectrum.

### The assimilation—integration spectrum

Concerning acculturation to the host culture in general, most of the interventions in the assimilation-integration spectrum tend to offer information (in the broader sense) that allows melting into and participating in the host culture (Birman et al., 2008; Block & Gibbs, 2017; Hála, 2017; Mwanri et al., 2012; Whitley et al., 2016/Whitley & Gould, 2011), sometimes combined with more or less distinct areas of home-culture maintenance (Birman et al., 2008; Mwanri et al., 2012). However, in all articles in this spectrum, the health-related acculturation strategy is directed at assimilating to a Western view of health with its focus on biomedicine and responsibilising individuals (Nettleton, 2013).

The “Evangelisches Jugend- und Fürsorgewerk” (EJF, a protestant organization for welfare and the young) represents an evangelical, native-driven organization with a longstanding tradition of charity work, based on a Christian commitment to helping the sufferer in need and a belief in “the gift of salvation” (Hála, 2017, p. e306) as well as the not-so-hidden agenda of keeping the social order intact (as exemplified by the “preventive criminological aspect” [Hála, 2017, p. e308] which Hála describes regarding one of the orientational projects). A selection of the EJF’s activities in Berlin is presented, which aim at helping “legal and legally-living immigrants” (Hála, 2017, p. e304) in different stages of introduction to German society. Among those are places providing accommodation and mental, medical and/or social stabilization in the first incoming phase as well as counselling and education in both the orientational second and the integrative third phase. Whereas second phase counselling and education focus on navigating Germany regarding employment, housing, language and study issues, third phase counselling and education are more focused on improving everyday life by means of courses in, for example, healthy living—Hála mentions an “exercise and healthy eating programme”—or by family education (e.g., in the course of a parent-child-group or coffee talks).

The range of therapeutic, social and educational activities and the demand that the target group has a legal status and acts in a legal manner reveals a clear focus on introducing the migrants to a new society in terms of giving them the means to blend in without disruption. Hence the EJF aims for assimilation as an acculturation strategy. In the intervention migrants are regarded in general as needy sufferers, highlighting therapeutic capacities for their mental stabilization and healthy living courses for securing their future health. Moreover, the initiative’s approach to health is clearly based on pathogenic and preventive thinking and grounded on the biomedical paradigm.

The African Communities Council of South Australia (ACCSA), a peak organization of African communities, (Mwanri et al., 2012) is an initiative that offers a range of different programmes (covering housing, law and social order and the like) that are mainly directed at participation in the host society but which also comprise activities oriented towards the maintenance of African cultures. The participation-oriented programmes are usually of an advisory or educative nature, handing out information and raising awareness of how things work in Australia (workplace rules, the rights and obligations of tenants and so forth), which might support assimilative strategies. In relation to health, ACCSA provides a series of different physical health programmes such as a “physical activity and nutrition programme”, “cervical awareness project” or a project against binge drinking, that reflect a Western health perspective and a preventive approach. Thus, as implemented programmes, they implicitly suggest an assimilation to this Western perspective.

There are a few interventions directed at home-culture maintenance and presentation and these include activities such as raising awareness in second-generation migrants of parents’ cultural values, African festivals or soccer tournaments for African communities. Even if most of them may carry a certain risk of turning “African cultures” into a showcase, they have the potential of balancing the assimilative tendencies and supporting integration. Thus, by offering such a range of programmes, the ACCSA implicitly proposes acculturation strategies that are located between integration and assimilation, yet which have a more pronounced focus on assimilation because of a health-related acculturation that predominantly serves an assimilative strategy.

The different sports programmes studied by Block and Gibbs (2017) included short-term and ongoing programmes directed at young people from a refugee background as well as integration into mainstream clubs provided by non-governmental organizations, local government, schools and both elite and grassroots sports clubs. The different organizers express a will to promote integration by mediating relevant values and skills through training so that the participants learn “to apply values and skills that they learned from playing a sport such as ‘working hard’, ‘persistence’ and ‘bouncing back from disappointment’ to other aspects of their lives” (Block & Gibbs, 2017, p. 95). As these values and skills show a bias towards representing a Western cultural imprint, the targeted integration can be regarded as showing clear assimilation tendencies even if an integration strategy may also be supported. This assimilative tendency is supported by health-related acculturation based on a Western view of health. This Western view of health is regarded as being represented by a perception of sports that is focused on learning objectives, with health promotion as one of its declared targeted benefits. It should, however, be noted that the way of organizing activities may sometimes promote separation tendencies if the offered sports community comprises “refugee-background” participants only.

The “Refugee Sports Club” (RSC) described both in Whitley and Gould (2011), and Whitley et al. (2016) is a short-term intervention comprising both discussions and reflections on a variety of values and their practice and realization in different sports activities. It is based on the “Teaching Personal Social Responsibility” (TPSR) model, which the authors adopt to promote healthy development. As the TPSR model is of Western origin, it can be regarded as representing a Western-style health concept. This model has been developed to help character building in underserved young people through sports with a focus in the RSC on mediating and enhancing respect, teamwork, leadership and the transfer of skills to everyday life. Discussing values may pave the way for both an integration-promoting exchange of notions regarding these values or for the adoption of a culturally opportune interpretation of those values, i.e., assimilation. Given the Western origin of TPSR or the idea of providing participants the opportunity to learn “American” sports, a tendency to promote assimilation into a melting pot appears to be more likely than integrating into a multicultural society.

The exercise programme described by Hashimoto-Govindasamy and Rose (2011) has been conducted alongside a broader resettlement support programme for Sudanese people in Australia, which is remotely outlined as following a community development approach and focussing on both group interests and cultural norms and strengths. On these vague grounds, the intervention as a whole may provide an opportunity for integrative or assimilative strategies. The selection of activities in the exercise programme, however, may be read as emphasizing assimilation rather than emphasizing integration. Although Sudanese children’s games are included as an element of the culture of origin, the programme exemplifies a Western-style health programme as it introduces physical activity and regular exercise (e.g., volleyball or Bollywood dancing) to counteract the effects of a sedentary resettlement lifestyle.

The programme “International Family, Adult, and Child Enhancement Services” (FACES) is a mental health programme with a comprehensive care perspective (Birman et al., 2008). Mental health symptoms and their consequences on the settlement process are presented as the intervention’s background and coping with and overcoming them is the goal of FACES. The programme comprises several different interventions and is provided by a multidisciplinary and multi-ethnic team. The team provides a broad range of resources and an understanding of cultural contexts while simultaneously carrying out work based on and oriented towards a Western frame of reference regarding health as the team ultimately aims for the application of traditional mental health services (such as counselling or occupational therapy). Given this culturally appropriate framing in combination with the provision of more traditional, Western-based mental health services, it can be assumed that FACES implicitly supports the assimilation-integration spectrum with an opening for integration because of the culturally-sensitive team.

Regarding the health dimension, there is a tendency to act on a pathogenic assumption. Five of the six interventions adhere to this perspective (Birman et al., 2008; Hála, 2017; Hashimoto-Govindasamy & Rose, 2011; Mwanri et al., 2012; Whitley et al., 2016/Whitley & Gould, 2011). As for practice, there are equally many interventions with a preventive focus (Birman et al., 2008; Hála, 2017; Mwanri et al., 2012) and with a promotive orientation (Block & Gibbs, 2017; Hashimoto-Govindasamy & Rose, 2011; Whitley et al., 2016/Whitley & Gould, 2011). Of the latter, however, only one intervention showed a distinct promotive strategy based on a salutogenic approach (Block & Gibbs, 2017). Moreover, it is notable that with one exception (Mwanri et al., 2012), all interventions, which have been sorted into this spectrum, are run by civil society organizations that are neither grassroots nor community-based but are primarily based on charity work.

### Integration

There are two interventions (Dhillon et al., 2020; Gonzalez et al., 2012) that distinctly point to integration as an acculturation strategy. The support group, “Parents Beyond Borders”, offers migrants a chance to explore their social identity as migrant parents, reduce their isolation and become empowered and capable of using the social services in the host society. The group is run with a pronounced focus on sharing immigration stories and experiencing empathy, validation and solidarity. Moreover, by discussing activism, participants are encouraged to “understand themselves as empowered agents of change in relation to larger entities such as (…) the community, and larger societal institutions” (Gonzalez et al., 2012, p. 26). These goals are considered to pave the way for the migrants’ home culture to change American ways and also embrace cultural opportunities of the existing host and a Western health perspective. As the programme stresses and encourages both possibilities simultaneously, it is regarded as implicitly suggesting a clear integration approach to acculturation. The programme’s goals reveal it as acting in a health-promotional manner based on a pathogenic understanding of its participants as vulnerable because of their immigration experience.

“Irene’s Project” is a recreational after-school club for young “convention refugees” with a focus on different forms of artistic, physical activity to foster self-efficacy, creativity and eventually acculturation (Dhillon et al., 2020). As it is introduced as “community education”, the project is presumed migrant-driven. The study focussed on a drumming circle as an inclusive environment and culturally responsive movement experience, which the refugees themselves had dedicated to that activity. “The space (…) became sacred to the newcomers, because it expressed a union between their existing culture and that of the host country (Canada)” (Dhillon et al., 2020, p. 321). The physical activity leader and the refugees sought to include and develop attendees’ repertoires of rhythms and moves in the drumming circle as an embodied, occupied space in which “social relations, cultural predispositions and the sense of self” are mediated and negotiated (Dhillon et al., 2020, p. 323).

In doing so, the interventions clearly promoted a general cultural integration and represented a salutogenic, resource-oriented approach. As physical activity is introduced and used in a playful, non-competitive and explorative way, its application is framed as contrasting to the biomedical paradigm with its focus on responsibilisation and goal-oriented production. This is highlighted in this article by the presentation of community physical activity programming as a culturally sensitive alternative to curriculum-based physical education.

### The integration-separation spectrum

The interventions that belong to this spectrum (Akinsulure-Smith & Jones, 2011; Gerber et al., 2017; Spaaij, 2012) are characterized by a more open view of culturally different understandings of health, which may resonate with acculturation strategies oriented towards integration or even separation. In the case described by Rich et al. (2015), the situation appears to be more intricate as its integration focus is accompanied by a tendency towards assimilation regarding acculturation in general and separation regarding health-related acculturation, which make integration appear to be a compromise between both of them.

The therapeutic and supporting programme of Nah We Yone (Akinsulure-Smith & Jones, 2011) serves forced migrants with a history of refugee trauma, war and human rights abuses. It provides a community-based empowerment model that promotes Western therapeutic practices by focusing on pan-African cultural strengths, in particular social and collateral ties and an awareness of the predisposition of African people to rely on traditional healers, elders and the like. An attempt is made to solve the participants’ difficulties within the community as African people are often reluctant to seek formal (Western) mental health services. Being aware of this situation, it is assumed that Nah We Yone has an open mind towards non-Western understandings of health and health procedures, which may promote the maintenance of the participants’ culture of origin while positioning these cultural understandings alongside Western understandings of health by offering certain therapeutic options. This programme will likely enable acculturation strategies that range from integration to separation as American and African culture is combined. However, the African basis appears to run somewhat deeper than the American superstructure.

The community-based Melbourne Giants Football Club (Spaaij, 2012) was established in the late 1990s to provide sporting opportunities to African refugees, particularly Somalis. Club activities are carried out by voluntary community residents and funded by local community organizations as well as by government and sport agency funds. The Melbourne Giants FC aims to enhance the well-being of the Somali community and culture, (re-)build internal (and, to a certain extent, external bicultural) social networks and develop leadership. As the promotion of social capital is presented as being much more important than promoting physical and mental well-being, the African cultural influence is apparent. The same applies to the choice of football (soccer) as a sports activity which, in Australia, is clearly played to a larger extent by migrants than by natives. The promotion of teamwork and leadership, in turn, resembles aims also highlighted by Western-influenced sports activities (see e.g., Whitley & Gould, 2011). In conclusion, the Melbourne Giants FC suggests an acculturation strategic spectrum that ranges from integration to separation, of which the latter appears to be more pronounced.

In the community gardening project located in the USA (Gerber et al., 2017), Nepali Bhutanese refugees were allowed to farm crops of their choice on multiple individual or family plots of earth as well as to cultivate shared crops on communal plots. The community gardens were provided and distributed by refugee resettlement agencies. These agencies consist of fifty-fifty international and national NGOs with religious, community-based or humanitarian backgrounds that act as operative partners of the US Division of Resettlement Services (Office of Refugee Resettlement, 2019). Gardening was intended to promote psychosocial health, well-being and support for a refugee group with pronounced mental health needs that are hard to accommodate with individual-oriented health services based on Western understandings of mental health. According to Gerber et al. (2017), community gardens function as hubs for exchanging information, getting social support, relaxation, shared identity work and feeling “as if (…) back in Bhutan” (Gerber et al., 2017, p. 25). The users often described experiences of independence and confidence about raising one’s voice, even when facing everyday challenges (such as employment issues or medical appointments).

On the one hand, the exchange of information and the experience of a creative gardening activity may contribute to an integrative acculturation strategy, which appears as confidence in everyday life. However, on the other hand, the facet of shared remembering and identity work indicates separative tendencies, with the gardens becoming a representation of a lost home in Bhutan and a place of withdrawal from the everyday reality in the US. Therefore, community gardens appeared to represent an acculturation strategic spectrum that ranges from integration to separation and may facilitate a movement in and out of American society. As for health, the choice of community gardening as an intervention acknowledges the Nepali Bhutanese refugees’ communal understanding of health, while working with a Western-style health vocabulary (mental health, well-being). This combination represents a pathogenic based, yet at the same time promotive oriented, US-established cultural health paradigm.

The “Community Cup” (Rich et al., 2015) soccer tournament combines both organizing and engaging in sports in a participatory manner. The shared event organization is carried out in teams that ideally include both newcomers and locals, framed by an organizational culture that is described as welcoming, friendly and non-judgemental. Simultaneously, a designated aim of the event organization is to provide newcomers with a Canadian experience and knowledge of working in their host culture, as this is deemed to be an asset on the employment market. The aim of working with people from different backgrounds is, therefore, combined with the experience of learning Canadian norms. Thus, newcomers may be directed towards either integration or assimilation-acculturation strategy.

Concerning health, the “Community Cup” both cites the Western idea of promoting health by promoting physical activity in the form of sports and a relational approach to health as belonging, which is often associated with migrant understandings. Given the form of the tournament with its focus on mediating an inclusive atmosphere instead of emphasizing competition connected to ideas of progressive (physical, mental) development, it may emphasize cultural understandings of health, related to the home society and not so much to the host society. It therefore paves the way for an integration and a separation strategy of health acculturation. This event-promoted way of organizing and engaging in sports may support a wide range of general acculturation strategies, depending on one’s self-assigned tasks. Here, however, integration can be defined as a kind of lowest common denominator. With its combination of health and general acculturation, the Community Cup has been deemed to belong to the integration-separation spectrum.

All but one (Gerber et al., 2017) of the interventions in the “integration-separation spectrum” group share a salutogenic background with three interventions that also implement a promotive practice (Gerber et al., 2017; Rich et al., 2015; Spaaij, 2012), whereas the activities of one intervention are preventive in nature (Akinsulure-Smith & Jones, 2011). If the two merely integration-supporting interventions are also considered, the tendency for resource-oriented health promotive actions at this end of the acculturation spectrum becomes even more distinct (even if Gonzalez et al., 2012 are based on a pathogenic background).

There is an openness about home-cultural understandings of health, or separation, in Berry’s terms (1997, 2008), in the “integration” as well as the “integration-separation spectrum” group, alongside an appreciation of the home culture in general. This may be no coincidence as two-thirds of the interventions (Akinsulure-Smith & Jones, 2011; Dhillon et al., 2020; Gonzalez et al., 2012; Spaaij, 2012) are run by community-based grassroots organizations. The majority’s view of (health) culture here can only be represented in a “second-hand” mode by those who have undergone similar acculturation processes to their intervention participants. The providers are therefore presumed to be more aware of the potentials and pitfalls of different cultural approaches and understandings but also more accepting concerning home-cultural understandings of health.

## Discussion

The main findings indicated that both the assimilation-integration and the integration-separation spectrums appear to be guided by implicit assumptions regarding three points of view: one on health, one on the participants and one on the kind of acculturation the host culture should be targeting.

Concerning the view on health, the mostly native-driven interventions in the assimilation-integration spectrum (Birman et al., 2008; Block & Gibbs, 2017; Hála, 2017; Hashimoto-Govindasamy & Rose, 2011; Mwanri et al., 2012; Whitley et al., 2016; Whitley & Gould, 2011) tend towards the notion that migrant groups inevitably and without questioning conform to their host culture’s health interpretations, or alternatively that other interpretations of health are not taken into consideration by the organizers as the Western hegemonic health view (Nettleton, 2013) prevails.

In the slightly more diverse, migrant-driven integration-separation spectrum (including the two interventions directed at integration only), the interventions appear to open up new spaces of intelligibility and practice concerning health, because they tended towards greater theoretical flexibility, ranging from biomedicine to a more collective view of health (Akinsulure-Smith & Jones, 2011; Dhillon et al., 2020; Gerber et al., 2017; Gonzalez et al., 2012; Rich et al., 2015; Spaaij, 2012). An openness concerning knowledge exchange and problematization of taken-for-granted “truths” has even been confirmed as a beneficial prerequisite for the enhanced accessibility of health care and, thus, an enhanced, co-produced migrant health (Barenfeld et al., 2015; Radl-Karimi et al., 2020).

Regarding the view of the interventions’ participants, migrant groups appear to be mostly framed as being either victims or risk carriers, i.e., people with problems, in the native-driven part and also the migrant-driven part of the assimilation-integration spectrum (Birman et al., 2008; Hála, 2017; Mwanri et al., 2012; Whitley et al., 2016; Whitley & Gould, 2011) as health-related actions usually aim to mitigate risks and (potential) suffering (with one exception, Hashimoto-Govindasamy & Rose, 2011).

In contrast to this, the health-related actions in the migrant-driven intervention of the integration-separation spectrum (Akinsulure-Smith & Jones, 2011; Dhillon et al., 2020; Gonzalez et al., 2012; Rich et al., 2015; Spaaij, 2012) appear to be based on a view of members of migrant groups as people with potentials (Antonovsky, 1996, 1993, 1979), as the salutogenic perspective is consistently emphasized (with one exception, Gonzalez et al., 2012). Moreover, Dahl et al. (2020, p. 1) point out that treating migrant groups “as a problem to deal with rather than as a possible resource for the society”, as implicitly manifested by applying a pathogenic approach, may be an attempt to protect the hegemony of one’s home (health) culture by a logic of controlling the stranger. Thus, a salutogenic, empowering approach may tend to correlate with better chances for integration than a pathogenic approach.

A community health perspective may be applied when bearing migrant groups in mind. This perspective reveals that the native-driven interventions may be described as, for the most part, using a top-down approach. In contrast, the majority of migrant-driven interventions appear to adhere to a bottom-up approach (Laverack & Labonte, 2000). However, talking about “communities” without further designations may result in missing the emphasis on acculturation, which questions the suitability of the community health perspective in the context of this study.

Moreover, concerning the kind of acculturation the host culture should be targeting, it is generally assumed that organizers—in comparison to participants—could be regarded as established members of society, even if the organizers are from a migrant background, which questions the idea of “one acting community” and spurs our adherence to the terms migrant/native-driven as well. The organizers’ ideas about acculturation describe points of view of the host culture. With this attribution in mind, the mostly native-driven interventions in the assimilation-integration spectrum (Birman et al., 2008; Block & Gibbs, 2017; Hála, 2017; Hashimoto-Govindasamy & Rose, 2011; Mwanri et al., 2012; Whitley et al., 2016; Whitley & Gould, 2011) could be described as being mainly directed at creating acculturation that ranges from melting pot to multiculturalism (Berry, 2008).

In contrast to this classification, the mostly migrant-driven interventions in the integration-separation spectrum (Akinsulure-Smith & Jones, 2011; Dhillon et al., 2020; Gerber et al., 2017; Gonzalez et al., 2012; Rich et al., 2015; Spaaij, 2012) are more focused on creating a spectrum that ranges from segregation to multiculturalism—or even a melting pot (Berry, 2008). Hence, different spaces of possibilities are implicitly or explicitly created in the assimilation-integration spectrum compared to the integration-separation spectrum.

These different spectral spaces entail different flexibility for practical and theoretical orientations for the participants during their acculturation, in terms of moving in and out of the host society and in-between different cultural traditions. Thus, the participants may be allowed, more or less, to conform with the host culture as a challenge for positioning themselves towards different cultural proposals in the essential identity work (Zacheus, 2010) and working for the often-endangered migrant health (Esses et al., 2017). Given that different types of host organizations mainly drive the interventions in the two spectra, this supports the conclusion of Osterlund and Seippel (2013) that different civil society organizations have different abilities to promote social inclusion. Moreover, engaging migrant-driven organizations in the integration task is considered of high significance. In doing so, the impact of the inevitable challenge to the very fabric of a person’s reality and identity, which migration is known to pose (Strandbu, 2005; Walseth, 2006; Zacheus, 2010), may be alleviated.

The challenging existential nature of the task at hand is indicated by the goals of the identified interventions (see, for example, the identity targeting focus on healthy development in (Whitley et al., 2016/Whitley & Gould, 2011) or the creation of new narratives on immigration and parenting as new ways of understanding reality in Gonzalez et al., 2012). Based on this existential nature, another consequence of the different ways of conceptualizing health-related integration interventions could occur with regard to processes of intercultural learning, which are undoubtedly relevant in the interventions (e.g., when jointly organizing a Community Cup [Rich et al., 2015] or establishing social networks beyond one’s own subculture while playing soccer in the Melbourne Giants FC [Spaaij, 2012]).

These processes include iterative circular movements between cultural naivety, shock and placeable understanding, resembling a hermeneutical circle between approaching and withdrawing from a culture with phases of curiosity, friction/conflict and the establishment of a new knowledge base (Scholten, 2001). Considering these intercultural learning processes, we cautiously suggest that the larger theoretical and practical spaces of possibilities, in combination with a potential-focused view of migrant groups—as provided by migrant-driven organizations—may facilitate the acculturation task for more members of migrant groups than the Western-focused, problem-oriented approach of native-driven organizations. In line with research which indicates a lower level of stress resulting from the experience of being linked to one’s cultural heritage and co-nationals, in real life or online (see, for example, Park et al., 2014), being able and encouraged to approach the host culture and temporarily withdraw into one’s home culture again to “take a break” before confronting oneself with a new aspect of the host culture may create spaces for reflectively processing one’s experiences, as well as breathing spaces characterized by the air of self-evidence connected to one’s home culture.

This could happen, for example, in the Melbourne Giants FC (Spaaij, 2012), and in the community gardens for the Nepali Bhutanese refugees (Gerber et al., 2017) or in the different branches of Nah We Yone (Akinsulure-Smith & Jones, 2011). Moreover, assuming that the separation strategy in the integration-separation spectrum is only applied temporarily may attenuate, even overcome, its adaptive disadvantage of being negatively oriented towards the host culture (compared to the double-positive orientation towards the home and host culture as implied by integration) and enhance its protective benefits. This may apply even more if the intervention participants are subject to prejudice and discrimination in the host society (Berry, 1997). However, the effect might depend on what individual members of migrant groups expect and require from their host culture.

In conclusion, the findings suggest a tendency for the acculturation strategies of migrant-driven organizations to be more oriented to integration, whereas the strategies of native-driven organizations are more oriented to assimilation. This tendency also recurs in the aimed-for health culture, where interventions conducted by migrant-driven organizations are characterized by openness and an understanding of broader perspectives of health. On the other hand, the strategies of native-driven organizations are more influenced by the health culture in the host society. The consequences of this are that an awareness of the basic assumptions about health and applied perspectives and strategies in health interventions is crucial for civil society organizations, based on the desired health and acculturation outcomes.

Given the expectations concerning interventions initiated by civil society organizations (Agergaard, 2011; Ambrosini & Van der Leun, 2015; Fredriksson et al., 2018; Schmidtke, 2018), the dearth of studies argues for more research into existing civil society interventions. As settings and migrant groups vary considerably, investigating specific settings and civil society organizations (e.g., the sports movement), or migrant groups (e.g., LGBTQ refugees) is perhaps to be recommended. In addition, the data collected did not permit an evaluation of the impact that understandings of health and acculturation may have on the “success” or “failure” of organizing health-integration interventions—and what kinds of impact are to be regarded as a “success” or a “failure”. To do so would be an additionally interesting task for future research.

In sharp contrast to the dearth of studies on civil society interventions, the research sector appears to take the lead when initiating, planning, placing and conducting interventions (see e.g., Koniak-Griffin et al., 2015; Kramish Campbell et al., 2007; Pirie et al., 2014; Rhodes et al., 2011). Our findings, however, imply and suggest that a reversal of this role allocation may be advisable in order to broaden the space of illegibility and practice (see also DeHaven et al., 2004), i.e., that (preferably migrant-driven) civil society organizations initiate health-related integration interventions, which could then be supported by research. The importance of collaboration to strengthen the social aspects of the integration of refugees and migrants is echoed in the work of Esses et al. (2017). Additionally, in line with the discussion above about agency and participatory processes (also found in Ferrera, 2017 study on integrating positive minority youth development with health promotion to empower an migrant community), we would like to stress the importance of participatory processes in health promotion with young migrants. Further, we suggest examining how the general health of young migrants is affected by taking part in integration and acculturation interventions by civil society, based on studies by Wimelius et al. (2017) and Bergström-Wuolo et al. (2018).

## Limitations

One limitation of the study was that we could only identify thirteen articles describing twelve different interventions that matched the inclusion criteria. This limited the possibility for us to draw generalizable conclusions so that our results may indicate tendencies but require further investigation. One possible way of overcoming this limitation would have been to include additional keywords, including broader terms such as *migrant* or *migration* or more specific terms regarding civil society such as *sports, church, non-governmental organization/NGO*, etc. Regarding the latter, we would, however, suggest that our chosen approach—a broad, general search followed by a selection concerning connections to civil society—resulted in more rather than fewer hits. Regarding the former, we cannot be certain. It appears, however, to be more likely that the number of identified studies rather indicates a general dearth of research conducted regarding the topic of health-related integration interventions by civil society organizations.

Another possibility to boost the number of interventions would have been to perform a scoping review (Armstrong et al., 2011), which allows the inclusion of grey material such as reports. However, as much as this probably would have increased the number of included interventions, it would not have provided an adequate overview of existing research.

It is, moreover, possible that we are biased and missed potential perspectives relevant to interpreting the data material due to prior knowledge. To tackle these potential sources of error, we repeatedly discussed the results of the study and its implications with the entire research team, which employs both specialists and newcomers in the field of integration and health research, as well as people with and without immigration experience in order to provide a broad spectrum of perspectives on the data.

The last concern may be the contrast between highlighted interventions originating from traditional “immigration countries” such as Canada, Australia or the USA (with only one study describing a German setting) and background papers that mostly represent a European setting. This may be a time-related side effect, reflecting the difference between long-standing experience and new demands in the wake of humanitarian motivated immigration movements mostly from the Middle East (cf. Eurostat, 2018). However, even if a complete congruence of results between different cultural settings cannot be achieved, we are optimistic that this study may still provide an orientation for those active in the field.

In sum, health-related civil society interventions are a rather unexplored field of research. The field offers a range of possibilities to develop such interventions in a participative way, under the lead of civil society organizations and to investigate their outcomes and hence answer the question of whether the expectations regarding their positive impact on migrants are justified.

## Data Availability

The dataset generated and analysed during the current study are available in the reference list (marked by *).
